# Relapse detection in the Danish surveillance program of patients with clinical stage I seminoma: a nationwide study

**DOI:** 10.2340/1651-226X.2025.42281

**Published:** 2025-01-31

**Authors:** Niklas B. Lindahl, Jakob Lauritsen, Thomas Wagner, Gedske Daugaard, Mikkel Bandak

**Affiliations:** a Department of Oncology, Copenhagen University Hospital, Rigshospitalet, Copenhagen, Denmark; b Department of Pathology, Herlev and Gentofte Hospital, Copenhagen University Hospital, Denmark

**Keywords:** Active surveillance, follow-up, germ cell cancer, seminoma, serum tumor markers

## Abstract

**Background and purpose:**

Active surveillance is a recommended management strategy for patients with clinical stage I (CSI) seminoma. This study aims to identify patterns of relapse detection methods in an unselected population-based cohort of CSI patients and provide evidence for a risk-adapted follow-up program.

**Patients/materials and methods:**

A total of 924 patients with CSI seminoma were identified in the prospective Danish Testicular Cancer database. Retrospectively collected clinical data were used for descriptive analyses of patterns in detection methods. Additionally, we explored a risk-adapted surveillance approach based on recently identified risk factors for relapse, classifying patients into low- and non-low-risk groups.

**Results:**

At 60 months, the 5-year cumulative relapse risk was 16%, with 146 relapses during surveillance. Relapses were detected by imaging alone in 71% of cases, imaging combined with elevated serum tumor markers (STMs) in 18%, isolated elevation of STMs in 8%, and by self-referral due to symptoms in 3%. No relapses were detected by abnormal findings at a physical examination. In total, 134 (92%) relapses were localized to retroperitoneal lymph nodes, primarily without additional spread. The 5-year relapse risk in patients with low risk of relapse was 9% compared to 28% in patients in the non-low-risk group.

**Interpretation:**

This study highlights that the surveillance program can detect relapses at an early stage. Reduction of visits and omission of routine physical examinations can safely be considered for patients with a low risk of relapse, while further research is needed to optimize follow-up and treatment for patients at higher risk of relapse.

## Background

Among men aged 15 to 44 years, testicular germ cell cancer (TGCC) is the most common malignancy in Western countries [1]. TGCC is histologically classified into seminomas and non-seminomas, with a slightly higher incidence of seminomas [2]. Approximately, 80% of patients diagnosed with seminoma present with testis-confined, clinical stage I disease (CSI) [3] and around 20% of these patients will develop a relapse after orchiectomy [4]. Furthermore, approximately 5% of men with TGCC develop cancer in the contralateral testicle, with two-thirds of these cases being metachronous tumors [5]. Today, the cure rate for patients with CSI seminoma approaches 100% [3].

The most effective management approach for CSI seminoma remains a topic of ongoing debate. International guidelines generally recommend active surveillance for patients at low risk of relapse, while adjuvant therapy, typically a single course of carboplatin, is considered an alternative to active surveillance for those at higher risk of relapse – with a strong emphasis placed on patient autonomy in the decision-making process [6–8].

Since 1984, Danish patients with CSI seminoma have been followed through a uniform 5-year active surveillance program, consisting of regular imaging (CT or MRI), measurement of serum tumor markers (STMs); lactate dehydrogenase (LDH), beta-human chorionic gonadotropin (β-hCG), and alpha-fetoprotein (AFP), and clinical visits with physical examinations [4]. Although modest changes have been made, a uniform and rigorous surveillance program is still applied to all CSI seminoma patients.

In recent years, the role of post-orchiectomy STMs [9, 10], routine physical examinations [11–13], and the extent of radiological scans [11] have been questioned. Furthermore, it has been suggested to modify follow-up based on the risk of relapse [14]. Despite this, a common risk-adapted surveillance program has not yet been established.

To our knowledge, Denmark is the only country that has followed all CSI seminoma patients nationwide with active surveillance in recent decades, providing us with the opportunity to evaluate relapse detection in a large, contemporary cohort of unselected CSI seminoma patients. Based on a complete review of patient records, the aim of this study was to clarify patterns in relapse detection methods and detection of metachronous contralateral TGCC as well as to explore the potential for risk-stratified surveillance programs, thereby securing a more evidence-based program capable of early relapse detection and avoiding unnecessary investigations.

## Patients and methods

We adhered to the Strengthening the Reporting of Observational Studies in Epidemiology (STROBE) guidelines for reporting [15].

This study included all men aged ≥ 15 years in Denmark diagnosed with incident CSI seminoma between January 1st, 2013 and December 31st, 2018. The study design has previously been described in detail [16].

The initial treatment was inguinal orchiectomy with a biopsy of the contralateral testicle to investigate possible germ cell neoplasia in situ (GCNIS) [17]. All patients were diagnosed with pure seminoma after a central pathology review. Post orchiectomy, patients were staged based on STMs and a CT scan of the thorax, abdomen, and pelvis. CSI disease was defined as no radiological or clinical evidence of regional or distant metastatic disease and normalized β-hCG post-orchiectomy. Patients with elevated STMs following orchiectomy were monitored weekly until normalization, confirming CSI. All patients were offered a uniform surveillance program performed at three university hospitals.

The Danish surveillance program of CSI seminoma is presented in Supplementary Figure 1 and includes 16 planned visits, each preceded by STM measurements. During the surveillance program, scheduled CT or MRI imaging was performed six times (months 6, 12, 18, 24, 36, and 60). Reproductive hormones were evaluated at 6, 24, and 60 months to identify patients with testosterone deficiency.

A complete medical record review was performed, obtaining information on date of birth, date of orchiectomy; radiological examinations, pre- and post-orchiectomy levels of STMs; clinical symptoms or findings at physical examinations; adherence; detection methods of relapse, confirmation of relapse, date, and location of relapse; emigration and death as per protocol; cause of death. Nationwide access to medical records secured follow-up information on internal migrants.

We defined the detection method of relapse as the following: (1) an abnormal routine radiological finding alone, (2) an abnormal routine radiological finding in combination with elevation of STMs, (3) elevation of STMs alone, (4) symptoms reported by the patient, or (5) an isolated abnormal finding at a routine physical examination. A confirmed relapse was defined as STM relapse (elevation of β-hCG) and/or radiological signs of metastatic disease and/or histologically verified relapse. The date of relapse was defined as the date of biopsy or surgery in the case of a histologically verified relapse. Relapses without histological verification were dated to the time of the initial detection method [16]. Relapses detected after the surveillance period were not within the scope of this study. Stage and risk classifications were based according to the American Joint Committee on Cancer (AJCC) and International Germ Cell Cancer Collaborative Group (IGCCCG) classifications.

Non-adherence to the surveillance program was defined as at least two missed follow-up appointments. In cases where it was unclear whether the criteria for non-adherence were met, the reviewing doctors reached a consensus.

Our group has recently published a population-based study on risk factors for relapse in patients with CSI seminoma in the same cohort of patients as this study [16, 18, 19]. In the study, tumor invasion into the testicular hilum – rete testis invasion (equivalent to one risk factor) and invasion into the hilar soft tissue (equivalent to two risk factors) – as well as lymphovascular invasion and elevated pre-orchiectomy levels of LDH or β-hCG (each one risk factor), were identified as independent predictors of relapse in CSI seminoma.

Based on these risk factors, we divided patients into a ‘low-risk group’ consisting of patients with the presence of 0 or 1 risk factor for relapse, where the estimated 5-year risk of relapse was 6–11%, and a ‘non-low-risk group’ with the presence of > 1 risk factor, where the estimated risk of relapse was 16–62% [18].

## Statistical methods

Descriptive statistics were used to examine the surveillance program and identify patterns in detecting relapses and other relevant aspects related to the surveillance program.

Follow-up was calculated from the date of orchiectomy to the date of death, relapse, loss to follow-up, or the last follow-up date. The risk of relapse was analyzed using the Kaplan–Meier method with relapse as an event and censoring at the time of emigration, death, diagnosis of metachronous contralateral TGCC, or study end date (July 20, 2022), whichever came first. The reverse Kaplan–Meier method was used to estimate the follow-up duration.

Patterns of relapse detection were evaluated in the whole cohort and stratified into ‘low-risk group’ and ‘non-low-risk group’.

Database management and statistical analyses were performed using SPSS (IBM SPSS Statistics for Windows, IBM Corp, Armonk, NY; version 29.0.1.0) and R (version 4.4.1).

## Results

The study included 924 patients with CSI seminoma, with a median age at diagnosis of 40 years. Additional baseline characteristics are shown in Table 1. At 60 months, the 5-year cumulative relapse risk was 16%, with 146 relapses detected during surveillance. The relapses were detected at scheduled follow-up visits in 141 cases (97%) and through self-referral due to symptoms in 5 cases (3%). In two patients, a relapse was detected after the end of the surveillance program – both approximately 7 years after the initial diagnosis, one with stage IIA and one with stage IIC. These two patients were not included in further analyses, as we focused on relapse patterns within the period of the surveillance program.

**Table 1:** Baseline characteristics of included patients.

	CSI seminoma total *n* = 924	
	No.	%
Age, years		
Median	40	
IQR	33–49	
Follow-up, years		
Median	6	
IQR	5–8	
Pre-orchiectomy STMs		
Normal STMs^a^	442	48
Elevated STMs	475	51
Unknown	7	< 1
Distribution of elevated STMs		
LDH alone	207	23
LDH and β-hCG	144	16
β-hCG alone	124	14

CSI: clinical stage I; IQR, interquartile range; STMs: serum tumor markers; LDH: lactate dehydrogenase; β-hCG: β-human chorionic gonadotropin.

a LDH ≤ ULN (depending on age) and β-hCG < 2 IU/L.

### Detection method and confirmation of relapses

**Figure 1:**
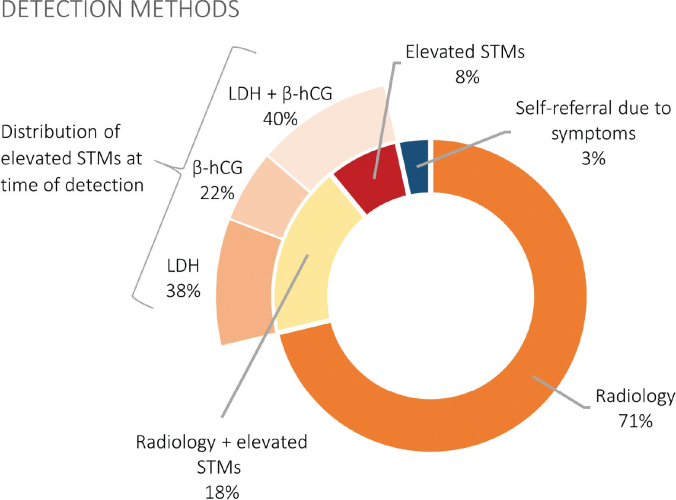
An overview of detection methods among the 146 relapses detected within the surveillance program and the distribution of elevated STMs. STMs: serum tumor markers; LDH: lactate dehydrogenase; β-hCG: β-human chorionic gonadotropin.

As shown in Figure 1, relapses were detected by abnormal findings on imaging alone in 71% of the cases, abnormal findings on imaging in combination with elevated STMs in 18%, and isolated elevation of STMs in 8%. In the remaining 3%, relapse was detected through self-referral due to symptoms outside scheduled follow-up visits. Thus, 89% of relapses were detected through imaging alone or in combination with elevated STMs. All relapses detected by isolated STM elevation were at scheduled follow-up visits that did not include planned imaging.

Three patients, in addition to the five who self-referred, experienced symptoms at the time of relapse. Five of the eight symptomatic patients reported a lump or discomfort in the inguinal region, two experienced back pain, and one noticed an abdominal mass. Two patients had objective findings on physical examination, presenting swelling in the groin area. No relapses were detected through physical examination without prior subjective symptoms, and no relapses were detected by AFP elevation.

In total, 79% of relapses were confirmed histologically, while the remaining 21% were verified through imaging, with or without elevated STMs. No relapses were verified by elevated STMs alone.

### Time to relapse

In total, 132 relapses (90%) occurred within the first 2 years of the surveillance program, while 14 relapses (10%) occurred more than 2 years after the initial diagnosis but still within the surveillance period.

**Figure 2:**
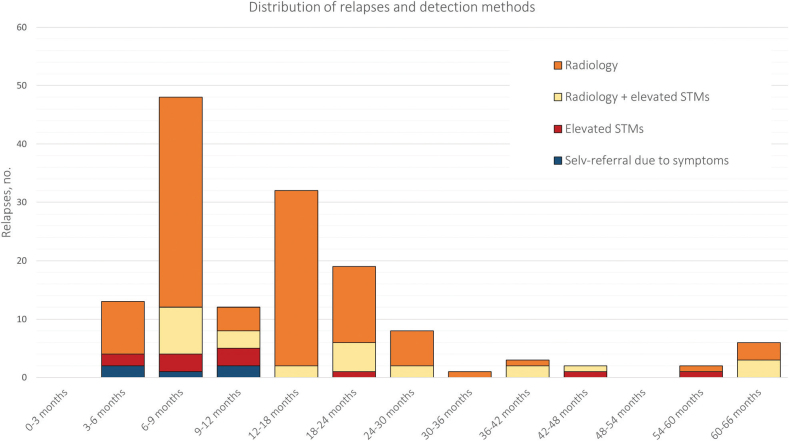
Distribution of relapses and their detection methods throughout the surveillance program: 3-month intervals in the first year, followed by 6-month intervals thereafter. STMs: serum tumor markers.

Figure 2 illustrates the relationship between the time to relapse and the detection method. Patients with isolated STM elevation were mainly identified during the first year of the surveillance program (8 out of 11). Furthermore, all five cases of self-referred relapses occurred within the first 2 years of the surveillance program.

Five patients developed metachronous contralateral TGCC, all with a contralateral biopsy without evidence of GCNIS. Two of these tumors were identified during the surveillance program, both as self-referrals with symptomatic testicular lesions; the remaining three were detected after the end of the surveillance program.

### Staging, prognosis, and adherence to the surveillance program

**Figure 3:**
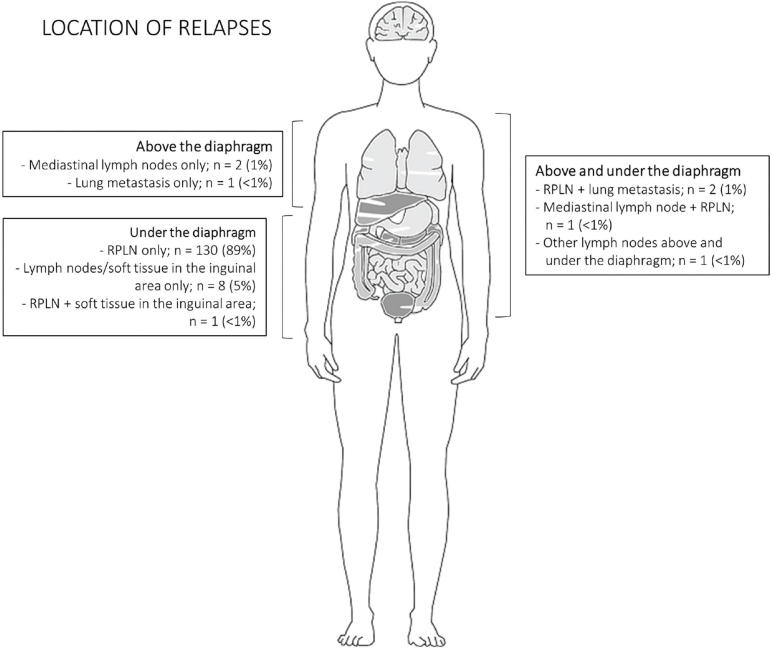
Location of metastatic disease at relapse. RPLN: retroperitoneal lymph nodes.

Of the 146 relapses detected during the surveillance program, 143 patients had metastatic disease below the diaphragm, of which 134 were localized to the retroperitoneal lymph nodes, primarily without additional spread (130 cases). Three relapses were localized solely above the diaphragm (Figure 3).

Of the seven patients with metastatic disease above the diaphragm (solely or concurrently with spread below the diaphragm), six relapses were detected within the first 2 years of the surveillance program and the last at the 36-month visit. There were no non-pulmonary visceral metastases. Detailed information about the stage and prognostic group is shown in Table 2. A total of 55% of relapses were treated with chemotherapy, 41% with radiotherapy, and 2% with surgery. Among the remaining cases (2%), two patients received a combination of chemotherapy and surgery, one received radiotherapy and surgery, and one elderly patient declined treatment. Three patients (0.3%) died of TC or due to complications to TC treatment.

**Table 2:** Characterization of relapses and detection methods.

Characteristics of relapses^a^	CSI seminoma total (*n* = 924)		Low risk (*n* = 480)		Non-low risk (*n* = 393)	
	No.	%	No.	%	No.	%
Number of relapses detected during the surveillance program^b^	146	16	40	8	102	26
Time to relapse, months						
Median	12		14		10	
IQR	7–20		7–21		7–17	
Time to relapse						
Year 0–2	132	90	35	88	94	92
Year 2–5	14	10	5	12	8	8
Detection methods						
Radiology	104	71	28	70	73	71
Radiology and elevated STMs	26	18	9	23	16	16
Elevated STMs	11	8	3	7	8	8
Physical examination alone	0	0	0	0	0	0
Self-referral due to symptoms	5	3	0	0	5	5
Distribution of STMs at time of detection						
Normal STMs^c^	109	75	28	70	78	76
Elevated LDH and β-hCG	15	10	3	8	12	12
Elevated LDH	14	10	5	12	8	8
Elevated β-hCG	8	5	4	10	4	4
Method of verification of relapses						
Histopathological verification	115	79	34	85	79	77
Imaging alone	24	16	5	13	18	18
Imaging and elevation of STMs	7	5	1	2	5	5
Staging (AJCC)						
IIA	82	56	23	58	56	55
IIB	47	32	13	32	33	32
IIC	7	5	3	8	4	4
IIIA	8	5	1	2	7	7
IIIB	1	< 1	0	0	1	1
IIIC	1	< 1	0	0	1	1
IGCCCG prognostic group						
Good	144	99	40	100	100	98
Intermediate	1	< 1	0	0	1	1
Poor	1^d^	< 1	0	0	1	1

CSI: clinical stage I; IQR: interquartile range; STMs: serum tumor markers; LDH: lactate dehydrogenase; β-hCG: β-human chorionic gonadotropin; AJCC: American Joint Committee on Cancer; IGCCCG: International Germ Cell Cancer Collaborative Group.

a Complete cases with information on all histopathologic variables were available for 873 patients. The remaining 51 patients, including 4 who experienced relapse, were not classified into the two risk groups.

b Two relapses were detected after the surveillance program ended: one in each risk group. As described in the text, they were not included in further analyses.

c LDH ≤ ULN (depending on age) and β-hCG < 2 IU/L

d One relapse was identified as a non-seminoma and due to an LDH level > 10 x ULN, the patient was classified in the poor prognostic group

In total, 63 patients (7%) were lost to follow-up during the surveillance program – the reasons were non-adherence in 28 patients, withdrawal by patient’s initiative in 15 cases, and moving abroad in 12 patients, while the reason was unknown in 8 patients. Two cases of relapse were detected by self-referral among patients who were lost to follow-up during the surveillance program.

### Risk stratification into ‘low- risk’ and ‘non-low-risk’ of relapse

A total of 873 patients had data on all four identified risk factors for relapse. As shown in Table 2, 480 patients were classified into the low-risk group (0 or 1 risk factors for relapse), and 393 patients were classified into the non-low-risk group (> 1 risk factor for relapse).

All relapses in the low-risk group (*n* = 40) were identified at a planned visit, and none of the patients had objective findings (Supplementary Figures 2 and 3). Table 2 presents a comprehensive overview of our analyses, including a comparison of relapse characteristics and detection methods between the two risk groups. A higher proportion of patients in the low-risk group had stage II disease (98% vs. 91% in the non-low-risk group). None of the relapses in the low-risk group were located solely above the diaphragm. The three patients who died of TC or TC treatment were in the non-low-risk group.

**Figure 4:**
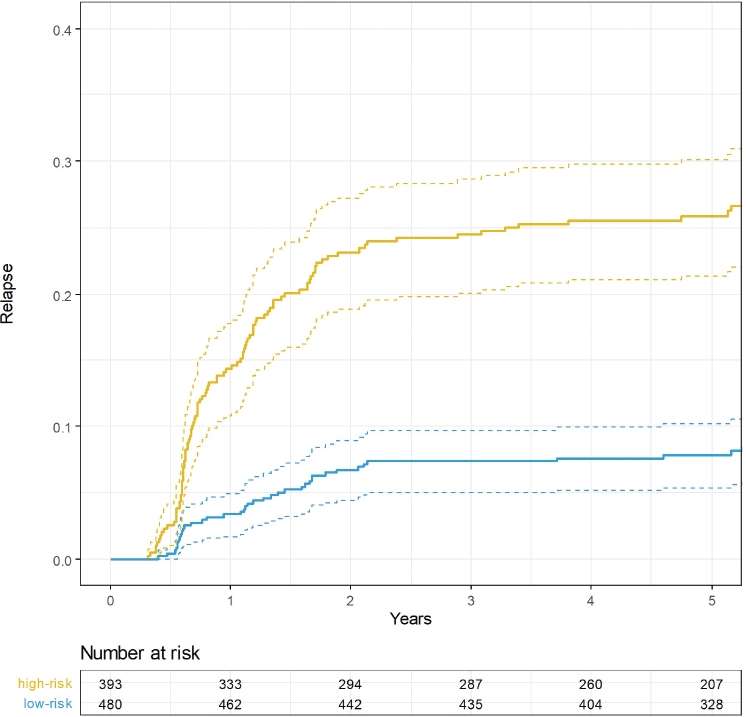
5-year cumulative risk of relapse stratified by risk group.

The cumulative risk of relapse after 2 years, 5 years, and overall was 7, 8, and 9% for patients in the low-risk group and 23, 26, and 28% in the non-low-risk group (Figure 4).

## Discussion

This study highlights the effectiveness of the Danish surveillance program for CSI seminoma, where the vast majority of relapses were detected in an early stage on a routine scan within the first 2 years. Nearly all relapses were curable and deaths due to relapse in CSI seminoma or its treatment were exceedingly rare. By dividing patients into low- and non-low-risk groups, we identified separate patterns of relapse, providing evidence for a risk-adapted follow-up program.

None of the patients in this study had their relapse suspected solely based on abnormal findings at a physical examination during a scheduled visit. This is in line with three other studies evaluating relapse detection in CSI seminoma patients, where no relapses were detected through physical examination alone [11–13], while it is contrary to a population-based Danish study including CSI seminoma patients treated from 1984 to 2007, where 5% of relapses were detected by abnormal findings at a physical examination [4].

However, limitations of the study include a retrospective study design with no clear definition of relapse detection, and we speculate that many of the abnormal findings at a physical examination were preceded by patient reported symptoms suggestive of a relapse. Furthermore, in this study, no metachronous testicular tumors were detected by physical examination.

In a high-tech society, it is possible to eliminate in-person visits and handle the reporting of blood tests and imaging results via phone or computer. However, it is important to emphasize that in-person visits serve purposes beyond relapse detection. For many patients, receiving a cancer diagnosis at a young age can be shocking and stressful [20]. The in-person visits provide an opportunity to explain and address questions on fertility, testosterone deficiency, heredity, relapse risk and treatment in case of relapse etc. In the current Danish surveillance program, there are 16 in-person visits. We do not advocate for the complete removal of visits and physical examinations; however, we suggest that the number of visits could be considerably reduced, especially for patients in the low-risk group. Routine physical examinations appear redundant and could be performed in case of symptoms. A reduction in in-person visits should lead to a greater focus on adherence, and it is necessary to consider a potential social bias regarding how effectively individual patients are at detecting symptoms of relapse and self-referring.

This study demonstrates that the measurement of STMs has relatively limited value in detecting relapses, as only 11 (8%) relapses were detected by isolated STM elevation. This finding aligns with the phase III noninferiority trial (TRISST), evaluating different imaging modalities in CSI seminoma. In TRISST, 10% of relapses were detected by isolated elevation of STMs.

There is inconsistency between international guidelines concerning the recommendation of routine evaluation of STMs in CSI seminoma as North American guidelines advocate against [21, 22], while European and Australian guidelines advocate for [7, 23]. A clear limitation of STMs, especially LDH, is the low specificity, with many false positive findings leading to unnecessary scans and stress for patients [24, 25]. Based on this study, it is not possible to make clear conclusions on the use of STMs, but the frequency of STM evaluations can possibly be reduced considerably in both the low-risk and non-low-risk group without compromising patient safety. As no relapses were detected based on AFP elevations, AFP can safely be omitted from the surveillance program. Currently, ongoing research aims to identify better biomarkers with greater sensitivity and specificity in the context of TGCTs. Here, microRNAs have emerged as promising biomarkers with the potential for improved diagnostic and early detection of relapse [26].

This study highlights the importance of scans, as the vast majority of relapses were detected through imaging findings. However, it is important to minimize unnecessary radiation exposure, as increased radiation at a young age has been associated with a theoretical risk of causing new cancers later in life [27], although the use of low-dose CT scans has already helped to reduce this risk.

In the TRISST trial, the use of CT versus MRI, as well as a reduced frequency of scans in the surveillance of CSI seminoma was evaluated [11]. The study showed that MRI is non-inferior to CT and that a 3-scan schedule (at time points 6, 18, and 36 months) was non-inferior to seven scans (at 6, 12, 18, 24, 36, 48, and 60 months) regarding clinical outcomes. A slightly higher proportion of stage ≥IIC relapses in the 3-scan groups was found, and 4/9 of these relapses would have been detected earlier with a 7-scan protocol. This is relevant, as patients with stage IIA or low-stage IIB (lymph nodes ≤3 cm) have other treatment options, including radiotherapy and retroperitoneal lymph node dissection [28–30]. Expanding MRI use could eliminate the radiation risks associated with CT scans but would require greater MRI availability and skilled radiologists for interpretation. A reduced scan frequency could also be considered for the low-risk group, where no relapses were detected by imaging between the 24-month and final 60-month follow-up visits (Supplementary Figure 2).

In our study, only three patients (2%) had isolated relapses above the diaphragm (one of these patients had concurrent hCG-elevation), and no patients in the low-risk group had relapses above the diaphragm. Most international guidelines do not recommend routine chest imaging as part of the surveillance program. Removing chest scans from the Danish surveillance program for CSI seminoma would significantly reduce radiation exposure for the total surveillance population and would eliminate the risk of false-positive findings (e.g. pulmonary/pleural nodules of < 1 cm interpreted as possible spread of germ cell cancer). On the other hand, it would also carry the risk that a minority of relapses may go undetected, leading to a higher stage at detection and increasing the risk of more complex treatment courses.

Compliance with the surveillance program was high, with only 7% of patients lost to follow-up during the surveillance program. Maintaining adequate compliance with the surveillance program is essential to avoid unnecessary late detections of relapses, which can affect staging, treatment options, and prognosis. Particularly when adjustments to the surveillance program are considered, ensuring a continued high level of compliance is crucial.

Two patients relapsed after the surveillance program. A more extended follow-up period would likely have revealed more late relapses, but another Danish study supports the rarity of relapses beyond 5 years [31]. However, it is important to emphasize patient education regarding relapse symptoms after completing the surveillance program.

A less intensive and simplified surveillance program should be considered for the low-risk group. Our findings suggest that physical examinations can be safely omitted, fewer STM measurements are sufficient, and chest imaging can be excluded from routine scans. Such adjustments could streamline the surveillance program, allowing physicians to allocate time to other priorities and reducing the burden of follow-up for patients. However, fewer scheduled visits would necessitate a stronger focus on compliance, targeted patient education on recognizing relapse symptoms, and easily accessible self-referral options.

For the non-low-risk group, additional studies are needed to support a more risk-adapted approach and identify a subgroup for whom adjuvant therapy may be considered, as adjuvant therapy is not without side effects and the efficacy of adjuvant carboplatin is relatively unsatisfactory [32, 33].

## Limitations and strengths

The primary strength of this study lies in its large, unselected, population-based setting, where all patients participated in a uniform surveillance program. No patients in the study population received adjuvant treatment, irrespective of histopathological characteristics. Centralized pathological review and standardized collection of relapse data minimized the risk of misclassification in our dataset.

A limitation of the study is its retrospective design, which may introduce biases in data collection and interpretation. We addressed this by using clearly defined variables and maintaining continuous dialogue among reviewing clinicians to ensure consistency in data collection. Also, the pathologists were blinded to the clinical outcome. Missing data were most pronounced when analyzing variables across the entire study population (e.g. compliance), whereas there were no missing values for variables related explicitly to relapse detection patterns in patients who experienced a relapse. Due to limited follow-up time for patients diagnosed with CSI seminoma toward the end of the study period, some relapses may have occurred post-data collection. However, since most relapses occur within the initial years following diagnosis, this limitation does not impact the interpretation of the study’s results.

## Conclusion

Most relapses in CSI seminoma patients on active surveillance are detected on routine imaging with or without elevated STMs. Routine physical examinations can be safely omitted as no relapses were detected by abnormal findings during these exams. For patients at low risk of relapse, the number of routine follow-up visits could be considerably reduced without compromising patient safety.

## Supplementary Material

Relapse detection in the Danish surveillance program of patients with clinical stage I seminoma: a nationwide study

## Data Availability

Due to data protection laws, researchers need to apply the Danish Health Data Authority to have access to the underlying person-level data.
