# Supplementary material for: Relapse detection in the Danish surveillance program of patients with clinical stage I seminoma: a nationwide study
Source: Acta Oncol. 2025 Jan 31;64:42281. doi: 10.2340/1651-226X.2025.42281 (PMC11811535; doi:10.2340/1651-226X.2025.42281)

Supplementary material has been published as submitted. It has not been copyedited, or typeset by Acta Oncologica

Supplementary figure 1: Standard 5-years surveillance program for CSI seminoma patients in Denmark Abbreviations: CSI, clinical stage I; CT, computed tomography; MRI: magnetic resonance imaging; LDH, lactate dehydrogenase; AFP, alpha fetoprotein; β-hCG, β-human chorionic gonadotropin; LH, luteinizing hormone

CSI seminoma	Month															
	2	4	6	8	10	12	15	18	21	24	30	36	42	48	54	60
In person visits with a physical examination	●	●	●	●	●	●	●	●	●	●	●	●	●	●	●	●
Blood tests	●	●	● (red)	●	●	●	●	●	●	● (red)	●	●	●	●	●	● (red)
Radiology (CT or MRI of the abdomen and thorax)			●			●		●		●		●				●

Blood tests: ● β-hCG, AFP and LDH, ● (red) β-hCG, AFP, LDH, total testosterone, free testosterone and LH

Supplementary figure 2: Distribution of relapses and their detection methods throughout the surveillance program in the low-risk group: 3-month intervals in the first year, followed by 6-month intervals thereafter Abbreviation: STMs, serum tumor markers

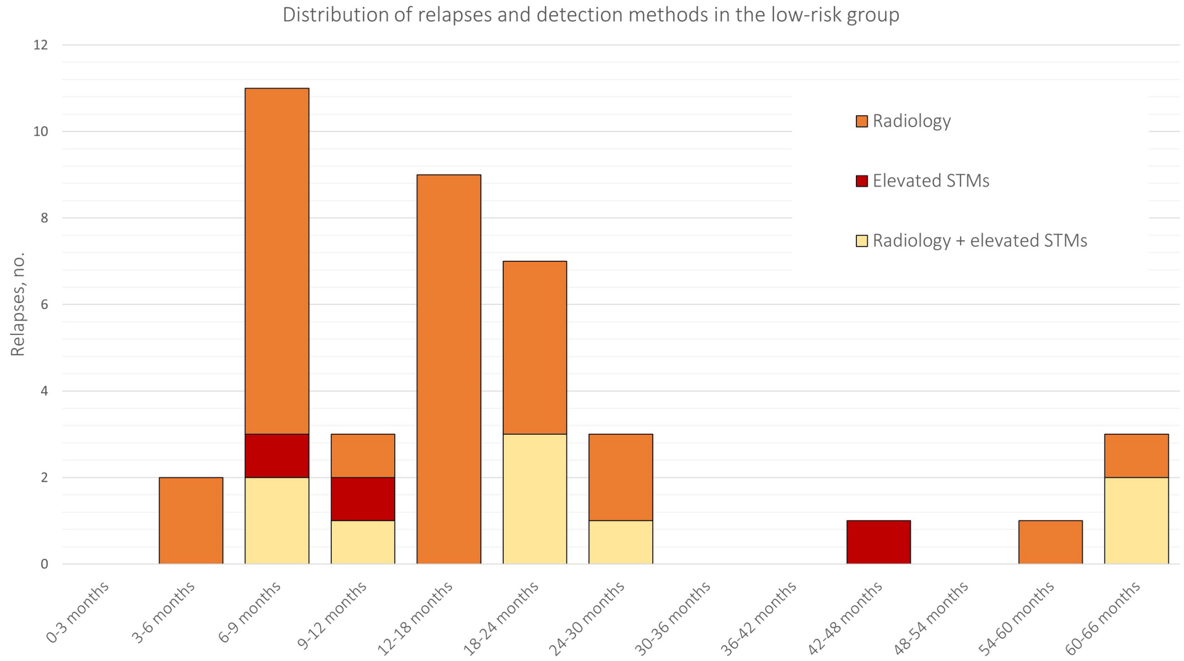

Supplementary figure 3: Distribution of relapses and their detection methods throughout the surveillance program in the non-low-risk group: 3-month intervals in the first year, followed by 6-month intervals thereafter

Abbreviation: STMs, serum tumor markers

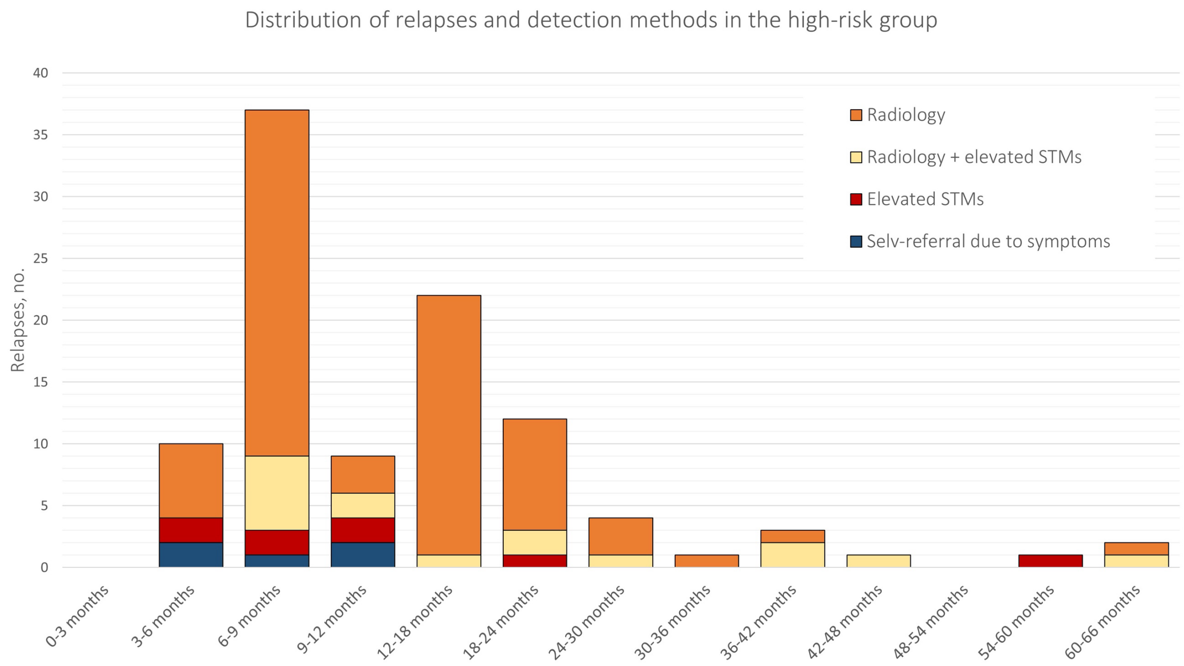

Supplement: Relapse detection in the Danish surveillance program of patients with clinical stage I seminoma: a nationwide study [file AO-64-42281-s1.pdf]
